# Impact of adverse childhood experiences on educational achievements in young people at clinical high risk of developing psychosis

**DOI:** 10.1192/j.eurpsy.2022.2351

**Published:** 2023-01-18

**Authors:** Stefania Tognin, Ana Catalan, Matthew J. Kempton, Barnaby Nelson, Patrick McGorry, Anita Riecher-Rössler, Rodrigo Bressan, Neus Barrantes-Vidal, Marie-Odile Krebs, Merete Nordentoft, Stephan Ruhrmann, Gabriele Sachs, Bart P. F. Rutten, Jim van Os, Lieuwe de Haan, Mark van der Gaag, EU-GEI High-Risk Study, Philip McGuire, Lucia R. Valmaggia

**Affiliations:** 1 Department of Psychosis Studies, Institute of Psychiatry, Psychology & Neuroscience, King’s College London, London, United Kingdom; 2 Outreach and Support in South London (OASIS) Service, South London and Maudsley NHS Foundation Trust, London, United Kingdom; 3 Biobizkaia Health Research Institute, Basurto University Hospital, OSI Bilbao-Basurto, University of the Basque Country UPV/EHU, Centro de Investigación en Red de Salud Mental (CIBERSAM), Instituto deSalud Carlos III, Barakaldo, Spain; 4 Department of Neuroimaging, Institute of Psychiatry, Psychology & Neuroscience, King’s College London, London, United Kingdom; 5 National Institute for Health Research (NIHR), Biomedical Research Centre (BRC), London, United Kingdom; 6 The National Centre of Excellence in Youth Mental Health, Orygen, Parkville, Victoria 3052, Australia; 7 Centre for Youth Mental Health, The University of Melbourne, Melbourne, Parkville, Victoria 3052, Australia; 8 Center for Neuropsychiatric Schizophrenia Research (CNSR) and Center for Clinical Intervention and Neuropsychiatric Schizophrenia Research (CINS), University of Copenhagen, Mental Health Centre Glostrup, Copenhagen, Denmark; 9 Faculty of Health and Medical Sciences, Department of Clinical Medicine, University of Copenhagen, Copenhagen, Denmark; 10 Faculty of Medicine, University of Basel, Basel, Switzerland; 11 LiNC - Lab Integrative Neuroscience, Depto Psiquiatria, Escola Paulista de Medicina, Universidade Federal de São Paulo – UNIFESP, São Paulo, Brazil; 12 Departament de Psicologia Clínica i de la Salut (Universitat Autònoma de Barcelona), Fundació Sanitària Sant Pere Claver (Spain), Centre for Biomedical Research in Mental Health (CIBERSAM), Madrid, Spain; 13 INSERM, IPNP UMR S1266, Laboratoire de Physiopathologie des Maladies Psychiatriques, Université Paris Descartes, Université de Paris, CNRS, GDR3557-Institut de Psychiatrie, Paris, France; 14 Faculté de Médecine Paris Descartes, GHU Paris - Sainte-Anne, Service Hospitalo-Universitaire, Paris, France; 15 Mental Health Center Copenhagen and Center for Clinical Intervention and Neuropsychiatric Schizophrenia Research, CINS, Mental Health Center Glostrup, Mental Health Services in the Capital Region of Copenhagen, University of Copenhagen, Copenhagen, Denmark; 16 Department of Psychiatry and Psychotherapy, Faculty of Medicine and University Hospital, University of Cologne, Cologne, Germany; 17 Department of Psychiatry and Psychotherapy, Medical University of Vienna, Vienna, Austria; 18 Department of Psychiatry and Neuropsychology, Faculty of Health, Medicine and Life Sciences, School for Mental Health and Neuroscience (MHeNS), Maastricht University, Maastricht, The Netherlands; 19 Department of Psychiatry, UMC Utrecht Brain Center, Utrecht University Medical Centre, Utrecht, The Netherlands; 20 Amsterdam UMC, Early Psychosis Department, Amsterdam, The Netherlands; 21 VU University, Faculty of Behavioural and Movement Sciences, Department of Clinical Psychology and Amsterdam Public Mental Health Research Institute, Amsterdam, The Netherlands; 22 Department of Psychosis Research, Parnassia Psychiatric Institute, The Hague, The Netherlands; 23 Department of Psychology, Institute of Psychiatry, Psychology & Neuroscience, King’s College London, London, United Kingdom

**Keywords:** Adverse childhood experiences, clinical high risk for psychosis, education

## Abstract

**Background:**

Adverse childhood experiences (ACE) can affect educational attainments, but little is known about their impact on educational achievements in people at clinical high risk of psychosis (CHR).

**Methods:**

In total, 344 CHR individuals and 67 healthy controls (HC) were recruited as part of the European Community’s Seventh Framework Programme-funded multicenter study the European Network of National Schizophrenia Networks Studying Gene–Environment Interactions (EU-GEI). The brief version of the Child Trauma Questionnaire was used to measure ACE, while educational attainments were assessed using a semi-structured interview.

**Results:**

At baseline, compared with HC, the CHR group spent less time in education and had higher rates of ACE, lower rates of employment, and lower estimated intelligence quotient (IQ). Across both groups, the total number of ACE was associated with fewer days in education and lower level of education. Emotional abuse was associated with fewer days in education in HC. Emotional neglect was associated with a lower level of education in CHR, while sexual abuse was associated with a lower level of education in HC. In the CHR group, the total number of ACE, physical abuse, and neglect was significantly associated with unemployment, while emotional neglect was associated with employment.

**Conclusions:**

ACE are strongly associated with developmental outcomes such as educational achievement. Early intervention for psychosis programs should aim at integrating specific interventions to support young CHR people in their educational and vocational recovery. More generally, public health and social interventions focused on the prevention of ACE (or reduce their impact if ACE occur) are recommended.

## Introduction

Adverse childhood experiences (ACE), including physical and emotional abuse and neglect and sexual abuse, are associated with the emergence of psychosis [1, 2] as well as with several cognitive deficits, including impaired memory, attention, emotional discrimination [3–5], and a decline in social and occupational functioning [6, 7]. Not surprisingly, ACE *per se* have been associated with worse educational attainments in both clinical and nonclinical populations [8–10]. Poor educational outcomes have been consistently associated with psychosis [11], as well as with several other mental health conditions. For example, autism spectrum disorder [12], attention-deficit/ hyperactivity disorder [13], and substance-use disorder [14] have been associated with low educational attainments. An exception is anorexia nervosa, which has been associated with high educational attainments [8].

Poor educational outcomes have been identified as one of the most significant long-term consequences of traumatic experiences in childhood [15]. One of the reasons is that poor educational attainment is linked to higher rates of unemployment later in life [10]. Possible mechanisms linking ACE to poor academic achievements are the neuropsychological changes that result from chronic childhood stress. For example, previous studies have shown how ACE can adversely impact the emotional stress response [16], social functioning [17], and cognitive functions [18], such as memory, attention and verbal learning, and, more generally, how ACE can interfere with early development [19].

A recent review and meta-analysis highlighted that all forms of ACE, including witnessing domestic violence, doubled the risk of school dropout [20]. Similarly, other studies reported that when individuals with ACE attend school, they are more likely to suffer bullying [21] and do less well in areas such as overall school performance and completion of tasks such as homework [22]. Poor educational attainments can have long-term repercussions on several areas, including mental health, financial security, and, more generally, needs to be met. For example, poor educational attainment is a risk factor for adolescent and adult difficulties, including conduct disorders [23] and substance abuse [24]. More generally, ACE might impact the achievement of formal qualifications, which in turn affects the likelihood of securing future employment [25, 26].

At their first contact with Early Intervention in Psychosis services (EIP), between 46 and 78% of the young people are not in employment, education, or training [27]. Adolescence and early adulthood are critical times to complete studies and enter the work environment and it is therefore important to investigate the impact that psychosis risk factors, such as ACE, might have on the vocational, including educational, pathway. To date, little is known about the impact of ACE on educational achievements in people at clinical high risk for developing psychosis (CHR). Despite ACE being highly prevalent in early psychosis, to the best of our knowledge, no prior studies directly examined the implications of ACE on educational achievements in the CHR population. In this study, we investigated the baseline associations between some of the ACE subtypes (i.e., emotional abuse and neglect, physical abuse and neglect, and sexual abuse) and education in a sample of CHR participants and a sample of healthy controls (HC) with similar sociodemographic characteristics. While the main focus of this work is education, for completeness we also analyzed at the baseline associations between ACE and employment. We hypothesized that (1) compared with HC, the CHR population will have completed fewer days in education and will have achieved lower levels of academic achievement and lower rates of employment. Moreover, (2) CHR and HC participants who experienced ACE will present worse educational outcomes (less days in education, lower level of education) and lower employment rates than those who did not. Finally, (3) the associations between ACE and educational outcomes will be more pronounced in CHR than in HC.

## Methods

### Sample

In total, 344 CHR participants and 67 HC were recruited as part of the European Network of National Schizophrenia Networks Studying Gene–Environment Interactions (EU-GEI) study [28] from 11 centers (London, Amsterdam, Den Haag, Vienna, Basel, Cologne, Melbourne, Copenhagen, Paris, Barcelona, and Sao Paolo) from July 2010 to August 2015. The overall design of the study was naturalistic, longitudinal, and prospective, consisting of a baseline and three follow-up time points when extensive clinical, cognitive, and biological measures were collected. CHR participants were recruited from local early detection for psychosis services, while HC participants with similar age, gender-ratio, ethnicity, and socioeconomic status were recruited from the same geographical areas. The inclusion criteria for all participants were as follows: being aged 15–35, being able to consent, and having adequate language skills local to each center. In addition, CHR participants had to meet clinical high risk for psychosis criteria defined according to the Comprehensive Assessment of At-Risk Mental States scale (CAARMS) [29]. The exclusion criteria for CHR participants were having had prior experience of a psychotic episode for more than 1 week as determined by the CAARMS and the Structural Clinical Interview for DSM Disorders (SCID); previous treatment with an antipsychotic for a psychotic episode; and an estimated intelligence quotient (IQ) < 60. Exclusion criteria for HC included meeting criteria for CHR or psychosis, reporting personal or (first-degree) family history of psychosis, and an estimated IQ < 60. The study received ethical approval at each site. All procedures contributing to this work comply with the ethical standards of the relevant national and institutional committees on human experimentation and with the Helsinki Declaration of 1975, as revised in 2008.

### Measures

#### Sociodemographics and clinical data

Detailed sociodemographic characteristics were assessed using the modified Medical Research Council Sociodemographic Schedule [28, 30]. The CAARMS [29] was used to measure CHR status and to determine the transition to psychosis. The SCID [31] was used to establish the presence of other psychiatric disorders and to exclude the presence of current or past psychotic disorders.

#### Adverse childhood experiences measures

The brief version of the Child Trauma Questionnaire (CTQ-B) [32] was used to measure ACE up to the age of 17. The CTQ is a 25-item self-report questionnaire that assesses five domains: emotional abuse, emotional neglect, sexual abuse, physical abuse, and physical neglect. A higher total score is related to a higher amount of adverse experiences.

Responses were used to calculate an individual’s total ACE score, which was then categorized into ACE counts (0, 1, 2–3, and 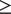 4), consistent with methodologies applied elsewhere [33].

Educational attainment was assessed using a semi-structured interview on years of education and level of education. The highest level of education was determined using six categories: (1) school, no qualifications—depicting those who had completed compulsory education but passed no exams, tests, and so forth to gain formal qualifications; (2) school, with qualifications—those who had completed compulsory education and passed one or more exams, tests, and so forth to gain formal qualifications; (3) tertiary, further—those with the first level of non-compulsory education, for example, A-levels or the International Baccalaureate; (4) vocational—those with job-related education, for example, teacher training, plumber, electrician, etc.; (5) higher (undergraduate)—those with a university degree; and (6) higher (postgraduate)—those with qualifications higher than first-degree level, for example, Masters or PhD. These categories were clustered into three variables: higher education (5–6), first-level non-compulsory education (3–4), and part-all compulsory education (1–2).

### Statistical analyses

Sociodemographic data were analyzed using means and standard deviation for continuous data and frequencies for categorical data. Analysis of the variance (ANOVA) was used to examine group differences in continuous variables. Chi-squares were used to assess differences in adverse experiences variables across groups and other categorical sociodemographic variables.

Linear (time of study measured as days) and multinomial (educational level-part-all compulsory education [reference category]) regression models were used to examine the relationship between variables of interest and ACE (as measured by the CTQ scale). We also performed binary regression models to examine the relationship between employment status (employed/not employed) and ACE. The following confounding variables were used to adjust the model results: gender, age, estimated IQ, social class, recruitment site, and ethnicity.

All tests were two-sided, and significance was set at *p* < 0.05. Analyses were carried out with the Statistical Package for the Social Sciences (SPSS) software program, version 24.0 [34].

## Results

### Descriptive analyses

In total, 411 participants were included in the analyses, 67 HC and 344 CHR. Sociodemographic characteristics of the samples are detailed in Table 1. At baseline, HC participants presented with higher estimated IQ, more years of education, lower rates of ACE, and higher rates of employment compared with CHR individuals. Approximately 52.6% of CHR compared with 18.2% of HC reported at least one ACE before the age of 17 (Table 2). This information was missing for 26 participants (1 HC and 25 CHR; 6.6%), who were therefore excluded from subsequent analyses.

**Table 1.** Sociodemographic and clinical and characteristics of the sample.

	CHR (*N* = 344)	HC (*N* = 67)	Statistics
Age in years mean (SD)	22.4 (4.9)	22.8 (4.1)	*t* = 0.8, df = 106.5, *p* = 0.4
Female *N* (%)	159 (46.2%)	33 (49.3%)	X^2^ = 0.21, df = 1, *p* = 0.37
Estimate of IQ mean (SD)	98.7 (16.2)	112.3 (17.9)	*t* = 5.8, df = 88.3, *p* < 0.001
Ethnicity white *N* (%)	246 (71.7%)	42 (66.7%)	X^2^ = 18.7, df = 5, *p* = 0.02
Childhood trauma total mean (SD)	47.5 (14.9)	34.8 (10.6)	*t* = −8.3, df = 112.7, *p* < 0.001
Emotional neglect	13.2 (4.8)	9.4 (4.7)	*t* = −5.9, df = 94.9, *p* < 0.001
Emotional abuse	11.9 (5.2)	7.4 (3.1)	*t* = −6.8, df = 383, *p* < 0.001
Physical neglect	8.2 (3.1)	6.4 (2.5)	*t* = −4.3, df = 383, *p* < 0.001
Physical abuse	7.2 (3.6)	5.9 (2.1)	*t* = −2.9, df = 383, *p* < 0.001
Sexual abuse	7.1 (4.2)	5.5 (1.9)	*t* = −3, df = 383, *p* = 0.003
Years in education mean (SD)	14.4 (3.1)	16.0 (2.8)	*t* = 102.3, df = 102.3, *p* < 0.001
Levels of education *N* (%)			
Part/all compulsory education	126 (41.7%)	19 (28.8)	X^2^ = 15.9, df = 2, *p* < 0.001
First-level non-compulsory education	133 (44%)	24 (36.4%)	
Higher education	43 (14.2%)	23 (34.8%)	
Employed *N* (%)	180 (59%)	60 (90.9%)	X^2^ = 24.2, df = 1, *p* < 0.001

**Table 2.** Number of ACE reported.

	> = 4 ACE	2–3 ACE	1 ACE	0 ACE
HC	1 (1.5%)	7 (10.6%)	4 (6.1%)	54 (81.8%)
CHR	29 (9.1%)	78 (20.3%)	74 (23.2%)	138 (43.3%)

Abbreviations: CHR, clinical high risk of psychosis; HC, healthy controls.

### Relationship between time in education and ACE

Higher CTQ total scores were significantly associated with fewer days in education in both the CHR sample (*R* ^2^ = 0.22; *F* [4, 272] = 20.725, *p* < 0.001; *B* = −9.3, SE = 4.2; *p* = 0.028) and the HC sample (*R* ^2^ = 0.49; *F* [3, 59] = 19.42, *p* < 0.001; *B* = –22.5, SE = 9; *p* = 0.015).

When different types of adverse experiences were analyzed separately, we found that emotional abuse was significantly associated with fewer days of education in the HC sample (*R* ^2^ = 0.50, adjusted = 0.48; *F* [3, 59] = 20.030, *p* < 0.001; *B* = −81.77, SE = 30.27; *p* = 0.009) but not in the CHR sample.

### Relationship between educational level and ACE

The level of education (higher education/first level noncompulsory education/part-all compulsory education**-**reference category) was significantly associated with CTQ total scores in the CHR group (odds ratio [OR] = 0.97, 95% confidence interval [CI] [0.95, 0.99]; *p* = 0.016, and OR = 0.94, 95%CI [0.9, 0.97]; *p* = 0.001) but not in the HC sample.

**Figure 1.**
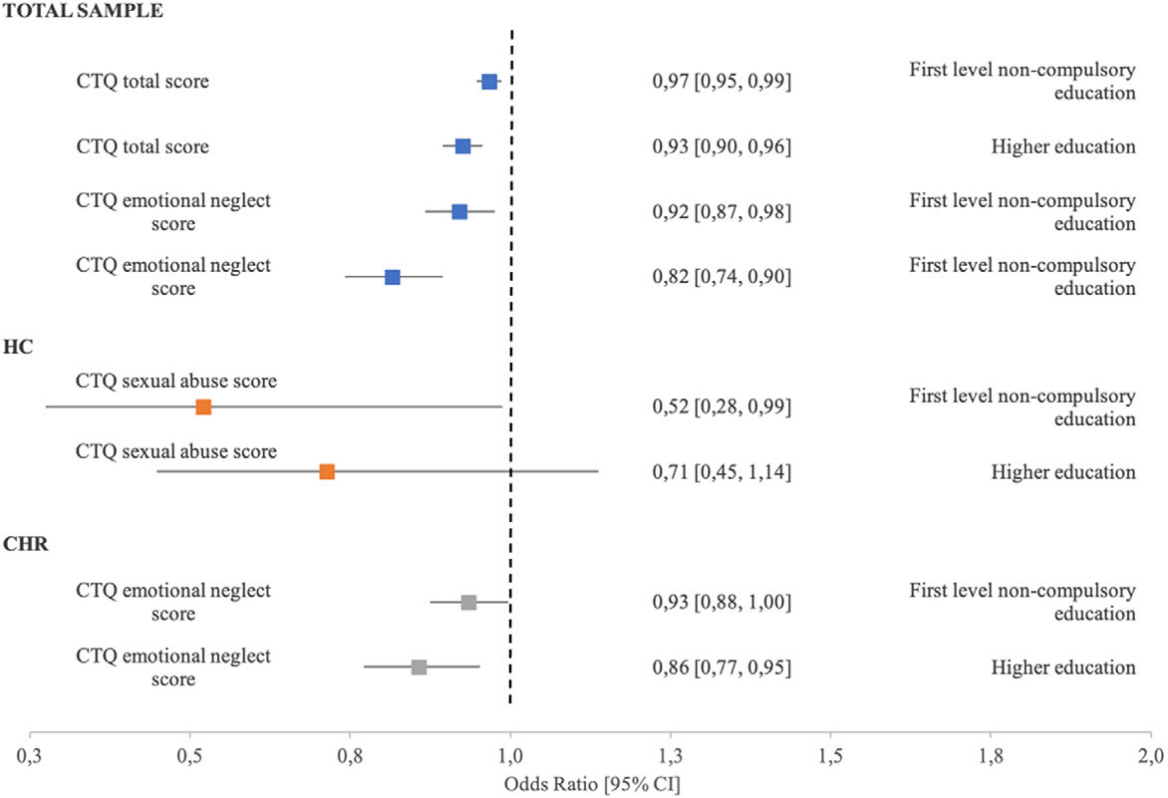
Adjusted odds ratios (adjOR) and 95% confidence intervals (CI) between CTQ scores and levels of education. Reference group: Part-all compulsory education.

When different types of adverse experiences were analyzed separately, we found that emotional neglect was significantly associated with lower rates of first-level noncompulsory education in the CHR group (OR = 0.92, 95%CI [0.90, 0.98]; *p* = 0.006), while in the HC group sexual abuse was significantly associated with lower rates of first-level noncompulsory education (OR = 0.52, 95%CI [0.27, 0.99]; *p* = 0.045) (Table 3 and Figure 1).

**Table 3.** Relationship between types of ACE and level of education.

CHR group								
Level of education		B	SE	Wald	df	*p*	Exp (B)	95%CI
First-level non-compulsory education	Intercept	−4.19	1.56	7.22	1	0.007		
	Age	0.23	0.04	27.01	1	< 0.001	1.25	[1.10, 1.5]
	Estimate of total IQ	0.02	0.01	5.41	1	0.020	1.02	[1.00, 1.03]
	CTQ emotional neglect score	−0.08	.030	7.55	1	0.006	0.92	[0.90, 0.98]
Higher education	Intercept	−15.03	2.61	33.22	1	< 0.001		
	Age	0.47	0.06	57.25	1	< 0.001	1.61	[1.42, 1.81]
	Estimate of total IQ	0.07	0.014	26.03	1	< 0.001	1.07	[1.04, 1.10]
	CTQ emotional neglect score	−0.20	0.05	18.12	1	< 0.001	0.81	[0.74. 0.9]

HC group								
Level of education		B	SE	Wald	df	*p*	Exp (B)	95%CI
First-level non-compulsory education	Intercept	−26.12	13.02	4.02	1	0.04		
	Age	0.93	0.41	5.05	1	0.02	2.53	[1.13, 5.68]
	Estimate of total IQ	0.07	0.044	2.68	1	0.102	1.07	[0.99, 1.72]
	CTQ sexual abuse score	−0.65	0.326	4.00	1	0.045	0.52	[0.27, 0.99]
Higher education	Intercept	−63.71	13.910	20.98	1	< 0.001		
	Age	1.29	0.427	9.13	1	0.003	3.64	[1.57, 8.41]
	Estimate of total IQ	0.125	0.050	6.33	1	0.012	1.13	[1.03, 1.25]
	CTQ sexual abuse score	−0.34	0.238	2.01	1	0.156	0.71	[0.45, 1.14]

*Note:* Reference category: part-all compulsory education.

Abbreviations: ACE, adverse childhood experiences; CHR, clinical high risk of psychosis; CTQ, Child Trauma Questionnaire; HC, healthy controls.

### Relationship between employment and ACE

In the CHR group, CTQ total score (OR = 0.98, 95%CI [0.96, 0.99]; *p* = 0.03), physical abuse (OR = 0.9, 95%CI [0.82, 0.98]; *p* = 0.025), and psychical neglect (OR = 0.82, 95%CI [0.73, 0.92]; *p* = 0.001) were significantly associated with unemployment. In the CHR group, emotional neglect (OR = 1.1, 95%CI [1.003, 1.15]; *p* = 0.04) was significantly associated with employment. No significant associations were found in the HC sample.

## Discussion

To the best of our knowledge, this is the first study to analyze the relationship between ACE and educational and vocational achievements in a large sample of CHR individuals and a sample of HC with similar sociodemographic characteristics.

Our first hypothesis was confirmed as our results showed that CHR individuals present significantly worse educational attainments, including fewer years in education than HC. Additionally, CHR individuals present significantly lower employment rates than HC. This is in line with previous studies reporting an association between most mental health conditions and poor educational achievement [8, 11–14]. For example, Cotter and colleagues [35] reported high rates of unemployment in a CHR sample (23%). At baseline, those who were unemployed at follow-up had a longer duration of untreated illness, more severe negative symptoms, lower IQ, poorer social and occupational functioning, and reported more childhood trauma than the employed group [35]. In our sample, 41% of CHR were unemployed during the baseline assessment. This high percentage is also consistent with previous reports [27, 36]. Taken together, these findings strengthen the idea that people in the early stages of psychosis generally experience severe difficulties with both education and employment.

Our second hypothesis was confirmed. Our results showed that high CTQ scores were associated with significantly fewer years of education in both the HC and CHR samples. In addition, emotional abuse was negatively associated with days of education in the HC but not in the CHR sample. These results carry significant clinical, public health, and societal implications as ACE are a modifiable and mitigable risk factor. When they come to the attention of Early Intervention Services, many young CHR individuals are already experiencing difficulties with education and employment [36]. While ACE seem to be affecting educational attainments in both HC and CHR individuals, CHR individuals are reporting overall significantly worse educational attainments than HC. The fact that ACE are similarly associated with educational attainments in both HC and CHR is important from a clinical point of view. HC with ACE might not come to the attention of clinical services, and yet they might require support to complete their studies. In this case, ACE screening and related interventions delivered within a school setting would be most appropriate. Schools are in a unique position to offer a non-stigmatizing intervention to support young people to complete their studies successfully [37, 38]. If the young person is already in contact with mental health services, interventions such as Individual Placement and Support with a dual focus on employment and education might be most appropriate [36].

Higher ACE scores were significantly associated with lower educational levels achieved in the CHR sample, but not in HC. In the CHR group, emotional neglect was significantly associated with lower educational achievements, while in the HC group, sexual abuse was associated with lower educational attainments. Interestingly, IQ did not significantly contribute to the prediction model up to the first level of compulsory education, but it contributed significantly to progressing to higher education. This supports the results of a previous study [10] where in those who achieved secondary level qualifications, ACE did not impact the progression to higher qualifications raising the possibility that completing the compulsory secondary level qualifications could mitigate some of the negative effects of trauma. Exposure to ACE in this sample has been previously described elsewhere [39], with higher rates of CTQ scores in the CHR sample compared to HC. Higher ACE rates in CHR individuals have been consistently described [40, 41]. ACE have also been associated with lower school attainment in clinical and non-clinical populations [10, 42, 43]. Chronic stress can negatively impact cognition, school connectedness, and school attendance [44], all of which may mediate the relationship between ACE and lack of educational achievement. In line with our results, Hardcastle and colleagues reported that positive history of childhood adversity doubled the risk of having no qualifications in a general population sample [10], suggesting a dose–response relationship as a higher number of ACE was related to poorer educational outcomes.

Finally, our study also showed some inconsistent findings with physical abuse and neglect associated with unemployment, in line with previous studies in the general population [10, 25]; emotional neglect associated with employment, rather than unemployment; and with the HC not being affected. Future studies should investigate additional factors such as ACE duration and family socioeconomic status to further clarify these relationships.

Our third hypothesis was partially confirmed. CHR individuals generally have lower educational attainments than HC. However, both CHR and HC are affected by ACE and our results indicate that HC are particularly affected in terms of missed days of education. Our findings suggest that HC who experienced ACE might miss a significant number of days in education (i.e., 22.5, and 81 when specifically affected by emotional abuse) while CHR still miss days, but less so (i.e., 9.3). This, however, needs to be put into context: while HC are missing more days compared to CHR individuals, they also spend overall more time in education (i.e., 16 vs 14.4 years) and achieve higher levels of education (14.2% CHR vs 34.8% HC complete higher education). Nonetheless, both HC and CHR are affected by ACE and should be provided with the appropriate support to overcome their difficulties, perform at their best and be able to complete their studies.

**Figure 2.**
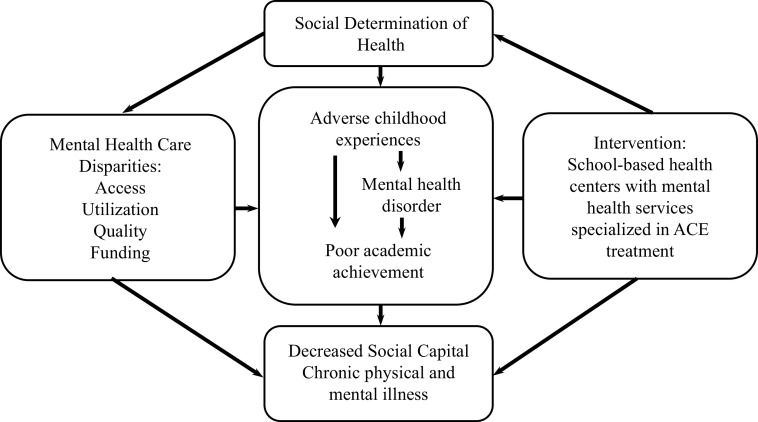
Conceptual framework. Adapted from Larson et al. [54]. Conceptual model created by Larson and colleagues that combined Link and Phelan’s 1995 social determinants of health, Braveman’s 2006 measurements of health disparities, and Felitti et al’s 1998 study of exposure to childhood adverse events and negative adult health outcomes [57–59].

This work examined the relationship between ACE and educational achievements. Most previous research on CHR individuals focused on the rate of transition to psychosis [45–47]. However, the majority of those at CHR of developing psychosis do not develop a psychotic disorder even after 10 years post-identification [48] while many remain symptomatic and functionally impaired [49]. Based on our findings, we propose that ACE influence academic achievement directly (Figure 2), and that related difficulties, such as symptoms of posttraumatic stress disorder, poor stress-management, low self-esteem, impairments in mentalization abilities, and interpersonal skills should be considered as a treatment target priority for children and adolescents [50], especially those more likely to present poor educational attainments, such as the CHR group. As the vast majority of ACE happen either within or close to the family, early family-centered therapeutic and educational interventions should be put in place to support the family as a whole as this is expected to both reduce ACE instances and to improve children’s and parents’ mental health [51]. These measures should also aim at targeting socioeconomic inequalities.

This work has several strengths. First, the sample is relatively large and very well characterized as all participants went through extensive and in-depth clinical interviews. Second, we were able to include an estimate of the IQ in our models, and we could therefore exclude that IQ uniquely explains educational attainments. This has important clinical implications in terms of the type and mode of intervention that can be offered to support pupils with mental health difficulties during school age.

This work has also several limitations. First, the HC sample is relatively small compared to the CHR one which might have limited the statistical power to detect differences. Secondly, even though the overall sample was large, when considering the different levels of education, we had to merge categories to allow sufficient representation. This might have affected the specificity of our results. Third, we were unable to investigate the relationship between educational attainments and transition to psychosis. This was due to the relatively low number of transitions to psychosis and the fact that not enough observations for all variables of interest (in particular when analyzing levels of education) were available.

Finally, although factors such as parental illness [52], parental socioeconomic status [53], duration of ACE [54], and migration are known to be linked with lower educational attainments, we could not explore these associations in this study due to a high number of missing data or to the information not being collected (i.e., duration of ACE).

Although a previous review highlighted that there is still a need for stronger and broader evidence base in the field of mental health promotion in young people within a school setting [55], a multidimensional and integrated approach to mental health promotion in this population is recommended [56]. In our opinion, this approach should include the appropriate and timely assessment of ACE and their potential impact on the young person’s life, including school attainments. This would ensure a holistic approach to mental health.

## Conclusions

The effects of ACE are associated with developmental outcomes such as educational achievement. From earlier research, we know that ACE are associated with an increased risk of psychosis [2]. This may generate two distinct sources of societal disadvantage and, in some cases, stigma. Public health and social interventions focused on prevention and early intervention around ACE are key [50]. Moreover, interventions to reduce the impact of ACE and to support vocational and educational achievements should be routinely implemented within early detection for psychosis services and in school settings.

## Data Availability

The data that support the findings of this study are available on request from the corresponding author. The data are not publicly available due to privacy or ethical restrictions.

## Supplementary material

For supplementary material accompanying this paper visit http://doi.org/10.1192/j.eurpsy.2022.2351. click here to view supplementary material
