# Supplementary material for: Impact of adverse childhood experiences on educational achievements in young people at clinical high risk of developing psychosis
Source: Eur Psychiatry. 2023 Jan 18;66(1):e16. doi: 10.1192/j.eurpsy.2022.2351 (PMC9970149; doi:10.1192/j.eurpsy.2022.2351)
Supplement: Supplementary file 1 [file S0924933822023513sup001.docx]

**Appendix**

**EU-GEI High Risk Study Group Author

Philip McGuire^1^

Lucia R. Valmaggia^24^

Maria Calem^1^

Mathilde Antoniades^1^

Sara Pisani^1^

Gemma Modinos^1^

Lieuwe de Haan^21^

Mark van der Gaag^22,23^

Eva Velthorst^21^

Tamar C. Kraan^21^

Daniella S. van Dam^21^

Nadine Burger^21^

Barnaby Nelson^7,8^

Patrick McGorry^7,8^

G Paul Amminger^7,8^

Christos Pantelis^25^

Athena Politis^26^

Joanne Goodall^26^

Anita Riecher-Rössler^11^

Stefan Borgwardt^11^

Erich Studerus^11^

Rodrigo Bressan^12^

Ary Gadelha^12^

Elisa Brietzke^12^

Graccielle Asevedo^12^

Elson Asevedo^12^

Andre Zugman^12^

Neus Barrantes-Vidal^13^

Tecelli Domínguez-Martínez ^27^

Manel Monsonet^28^

Lídia Hinojosa^28^

Anna Racciopi ^28^

Thomas R. Kwapil ^29^

Mathilde Kazes^14,15^

Claire Daban^14^

Julie Bourgin^14^

Olivier Gay^14^

Célia Mam-Lam-Fook^14^

Marie-Odile Krebs^14^

Dorte Nordholm^16^

Lasse Randers^16^

Kristine Krakauer^16^

Louise Glenthøj^16^

Birte Glenthøj^16,30^

Merete Nordentoft^16^

Stephan Ruhrmann^17^

Dominika Gebhard^17^

Julia Arnhold^17^

Joachim Klosterkötter^17^

Gabriele Sachs^18^

Iris Lasser^18^

Bernadette Winklbaur^18^

Harald Aschauer^18^

Philippe A Delespaul^19^

Bart P. Rutten^19^

Jim van Os^1,19^

^25^ Center for Neuropsychiatric Schizophrenia Research (CNSR) and Center for Clinical Intervention and Neuropsychiatric Schizophrenia Research (CINS), University of Copenhagen, Mental Health Centre Glostrup, Copenhagen, Denmark

^26^ University of Copenhagen, Faculty of Health and Medical Sciences, Dept. of Clinical Medicine, Copenhagen, Denmark

^27^ Global Mental Health Research Center, Directorate of Epidemiological and Psychosocial Research, ‘Ramón de la Fuente Muñiz’ National Institute of Psychiatry, Mexico.

^28^ Departament de Psicologia Clínica i de la Salut (Universitat Autònoma de Barcelona).

^29^Department of Psychology, University of Illinois at Urbana-Champaign (USA).

^30^ University of Copenhagen, Faculty of Health and Medical Sciences, Department of Clinical Medicine, Copenhagen, Denmark
